# Genome-wide analysis of gene regulation mechanisms during Drosophila spermatogenesis

**DOI:** 10.1186/s13072-018-0183-3

**Published:** 2018-04-02

**Authors:** Petr P. Laktionov, Daniil A. Maksimov, Stanislav E. Romanov, Polina A. Antoshina, Olga V. Posukh, Helen White-Cooper, Dmitry E. Koryakov, Stepan N. Belyakin

**Affiliations:** 1 Institute of Molecular and Cellular Biology SB RAS, 8/2 Lavrentyev Ave, Novosibirsk, Russia 630090; 2 School of Biosciences, Cardiff University, Cardiff, CF10 3AX UK; 3 Novosibirsk State University, Novosibirsk, Russia 630090

**Keywords:** Drosophila, Spermatogenesis, Gene regulation, DamID

## Abstract

**Background:**

During Drosophila spermatogenesis, testis-specific meiotic arrest complex (tMAC) and testis-specific TBP-associated factors (tTAF) contribute to activation of hundreds of genes required for meiosis and spermiogenesis. Intriguingly, tMAC is paralogous to the broadly expressed complex Myb-MuvB (MMB)/dREAM and Mip40 protein is shared by both complexes. tMAC acts as a gene activator in spermatocytes, while MMB/dREAM was shown to repress gene activity in many cell types.

**Results:**

Our study addresses the intricate interplay between tMAC, tTAF, and MMB/dREAM during spermatogenesis. We used cell type-specific DamID to build the DNA-binding profiles of Cookie monster (tMAC), Cannonball (tTAF), and Mip40 (MMB/dREAM and tMAC) proteins in male germline cells. Incorporating the whole transcriptome analysis, we characterized the regulatory effects of these proteins and identified their gene targets. This analysis revealed that tTAFs complex is involved in activation of *achi*, *vis*, and *topi* meiosis arrest genes, implying that tTAFs may indirectly contribute to the regulation of Achi, Vis, and Topi targets. To understand the relationship between tMAC and MMB/dREAM, we performed Mip40 DamID in tTAF- and tMAC-deficient mutants demonstrating meiosis arrest phenotype. DamID profiles of Mip40 were highly dynamic across the stages of spermatogenesis and demonstrated a strong dependence on tMAC in spermatocytes. Integrative analysis of our data indicated that MMB/dREAM represses genes that are not expressed in spermatogenesis, whereas tMAC recruits Mip40 for subsequent gene activation in spermatocytes.

**Conclusions:**

Discovered interdependencies allow to formulate a renewed model for tMAC and tTAFs action in Drosophila spermatogenesis demonstrating how tissue-specific genes are regulated.

**Electronic supplementary material:**

The online version of this article (10.1186/s13072-018-0183-3) contains supplementary material, which is available to authorized users.

## Background

**Fig. 1:**
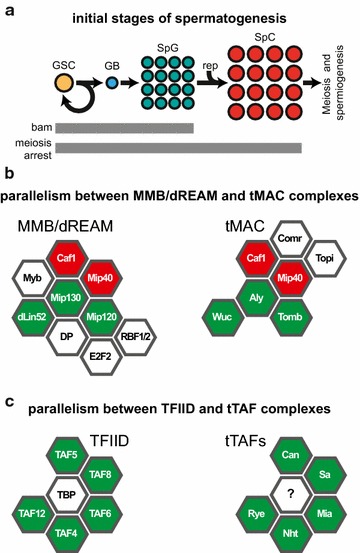
Drosophila spermatogenesis and the main regulators of gene activity. **a** An overview of the first stages of spermatogenesis in Drosophila. Germline stem cell (GSC) divides asymmetrically producing a gonioblast (GB). After four mitotic divisions, a cyst of 16 spermatogonia (SpG) is formed. These differentiate synchronously to spermatocytes (SpC) that replicate their chromosomes and enter meiosis. Mutation in *bam* gene precludes the differentiation step resulting in accumulation of SpG cysts in the testis. Meiosis arrest mutants fail to proceed to meiosis and accumulate SpC cysts. Gray bars indicate the germline cell types that are presented in the testes of *bam* or meiosis arrest mutants and contribute to the DamID and expression profiling experiments in this study. **b** Comparison of tMAC and MMB/dREAM complexes. Two complexes share common components (red) and contain homologous subunits (green). **c** Comparison of tTAFs and TFIID complexes. Homologous subunits are shown in green. TBP protein associated with tTAFs is unknown

During differentiation, cell identity switches from proliferating stem cell to a specialized cell with distinct physiological function. This process involves several mechanisms that ultimately converge on the activation of genes required for future function of the cell and on the repression of genes that are needed for stemness or function in other cell types. Here, we used Drosophila spermatogenesis as a model to study the mechanisms of gene activation during cell differentiation (Fig. 1a). When a male germline stem cell divides, one of the daughter cells becomes a gonioblast, which undergoes four mitotic divisions forming a cyst of 16 spermatogonia that differentiate into spermatocytes and enter meiosis. Meiotic program and subsequent spermiogenesis require massive activation of many genes, of which about 1500 are expressed only in spermatocytes, as inferred from the whole genome microarray data [1, 2].

Differentiation of spermatogonia into spermatocytes depends on the *bag of marbles* (*bam*) gene (Fig. 1a), and mass activation of genes in spermatocytes requires two classes of spermatocyte-specific transcription factors encoded by *meiosis arrest* group of genes. Mutations in these genes result in meiosis arrest in the G2 that precedes meiosis I (Fig. 1a) and lead to accumulation of mature primary spermatocytes [3].

Meiosis arrest genes encode the components of two distinct protein complexes. Meiosis arrest complex (tMAC) [4] includes: Aly (Always early), Comr (Cookie monster), Topi (Matotopetli), and Tomb (Tombola), along with Mip40 (Myb-interacting protein 40) and CAF1-55 (Chromatin assembly factor 1, p55 subunit). These proteins form testis-specific assembly that shares several homologous subunits with the MMB/dREAM complex (Fig. 1b). Other proteins can be involved in tMAC, and their combinations suggest that there may be several tMAC-related complexes [5–7].

Can (Cannonball), Sa (Spermatocyte arrest), Rye (Ryan express), Mia (Meiosis arrest), and Nht (No hitter) are testis-specific homologues of TBP-associated factors (tTAFs) that probably form a testis-specific paralogue of TFIID (Fig. 1c) [8, 9]. It was previously reported that mutations in tMAC components show dramatic decrease in expression of about 1000 genes; mutations in tTAFs fail to activate about 350 genes, most of which also depend on tMAC [3].

Previous studies suggested that Polycomb complexes play a central role in repressing spermatocyte-specific genes in undifferentiated precursors [2, 10]. This model, however, has been recently challenged in a genome-wide study that failed to detect association of Polycomb with the promoters of testis-specific genes in spermatogonia [11]. One of the alternative mechanisms of spermatocyte-specific genes repression in spermatogonia may involve MMB/dREAM activity, as this complex has been shown to function as a repressor [12–14]. In this regard, similarity between tMAC and MMB/dREAM raises the interesting possibility that these complexes interact to regulate spermatocyte-specific gene program. To complete the picture of gene regulation in spermatogenesis, a new mechanism, involving Kmg and dMi-2, that prevents the expression of the somatic genes in Drosophila male germline was recently discovered [15].

Here, we investigated the binding of tMAC and tTAFs components to the chromosomes and studied their effects on transcription. Specifically, we performed germline cell-specific genome-wide profiling of the Cookie monster (Comr) protein representing tMAC, Mip40, which is a subunit shared by tMAC and MMB/dREAM, and Cannonball (Can, tTAF). Our study revealed the mutual dependencies between these factors that provide the new aspects in regulation of tissue-specific genes.

## Results

### Germline-specific genome-wide DamID analysis of Comr, Can, and Mip40 identifies their cognate target genes

 Despite the fact that mutations in tMAC and tTAFs subunits cause down-regulation of hundreds of genes, very little is known about their direct gene targets. Only three gene targets of the Spermatocyte arrest protein (Sa, tTAF) have been reported in the literature and include *dj* (*don juan*), *fzo* (*fuzzy onion*), and *Mst87F* (*Male*-*specific RNA 87F*) genes [2, 10].

We used tissue-specific DamID-seq to establish the genome-wide profiles of Cookie monster (Comr, tMAC subunit), Cannonball (Can, tTAFs subunit), and Mip40 (shared between tMAC and MMB/dREAM complexes) specifically in the *D. melanogaster* male germline [16–18]. Comr and Can are essential components of tMAC and tTAFs [8, 19, 20]. In our previous paper [17], we demonstrated direct activating role of Comr in spermatocytes and performed the initial characterization of the interplay between Comr and Can. The present study improves that analysis with higher resolution and sensitivity and allows to uncover the new aspects of regulatory events in Drosophila spermatogenesis.

**Fig. 2:**
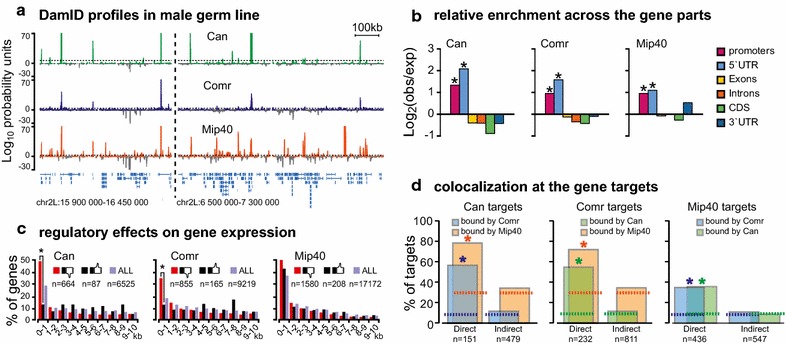
DamID profiling of Can, Comr, and Mip40 in the testes of wild-type male flies and identification of Can, Comr, and Mip40 gene targets. **a** DamID profiles for Can, Comr, and Mip40 proteins in the germline cells of wild-type males. Peak height corresponds to the value of − log_10_(*P*), where *P* is the significance value (*P*-value) measured using Fisher’s exact test (log_10_ probability units). Peaks above the *x* axis correspond to the regions enriched with the protein of interest, and peaks below the *x* axis denote genomic regions depleted for this protein. Dashed line shows the significance threshold for peak calling that corresponds to a FDR = 0.05. **b** Analysis of genomic distribution of protein localizations compared to the random distribution in a set of testis-specifically expressed genes. All three proteins tend to localize to gene promoters and in 5′-UTRs (asterisks—binomial test *P* < 10^−3^). **c** Analysis of the interplay between Can, Comr, and Mip40 binding and gene activity. RNA-seq analysis was used to assess gene expression in the testes of *can*, *comr*, and *mip40* mutants versus wild-type testes. Transcripts showing greater than fourfold difference in gene expression were used in the analysis. For each transcript, the distance between its TSS and the closest Can/Comr/Mip40 peak was calculated and plotted in 1 kb bins within 10 kb around the TSS. Asterisks indicate that the differences among the groups of transcripts showing greater than fourfold changes in expression are statistically significant (Chi-square test, *P* = 1.8 × 10^−10^ in the case of Can and *P* = 5.7 × 10^−5^ in case of Comr). Differences were insignificant for Mip40 gene targets. **d** Gene targets tend to be cooperatively bound by the proteins studied. This is not the case for indirectly controlled genes. Asterisks denote significant differences (Chi-square test, *P* < 3.6 × 10^−7^ in all cases), dotted lines—randomly expected values

Testes from 3-day-old wild-type adult males were used for this analysis, as they express the normal profiles of the tested proteins throughout the spermatogenesis. DamID-derived libraries were subjected to Illumina sequencing. Data analysis, generation of profiles, and identification DamID peaks were based on the algorithm [18] described in the Methods. DamID signals are presented as − log_10_(*P*), where *P* is Fisher’s exact test *P*-value calculated for each genomic fragment to estimate the difference between the samples expressing Dam-fused transcription factor versus Dam-alone control [18] (log_10_ probability units in Fig. 2a). Positive values represent genomic regions enriched with the Comr, Can or Mip40, and negative values designate the regions that are relatively depleted with the proteins of interest.

Peak calling pipeline identified 2140 significantly bound peaks for Can, 5422 peaks for Comr, and 12,981 peaks for Mip40. The following criteria were used for peak calling: FDR<0.05, significance threshold value *P* < 10^−3^, and log_2_(Dam-*X*/Dam) > 1 (where *X* stands for one of the proteins mapped, see Methods). Applying these criteria allowed us to detect the most prominent peaks for each Can, Comr or Mip40 (Fig. 2a), which is needed for reliable identification of genes that are under direct control of each protein. The difference between peak numbers was accounted for by the downstream statistical tests so as to calculate the expected threshold values. Genome-scale analysis of peak positions indicated that the colocalization of three proteins is much higher than randomly expected, as assessed with binomial test (Additional file 1: Fig. 1). The Euler diagram describing the intersection of detected peaks (Additional file 2: Fig. 2) shows that despite the significant overlap between the sets of binding sites, considerable amount of stand-alone peaks of Can, Comr, and Mip40 was observed.

Mip40 is a component of tMAC and its absence could affect the distribution of other proteins of this complex, including Comr. On the other hand, putative DNA-binding domain present in Comr protein could ensure its binding to the chromosomes independently from other tMAC components. We performed Comr DamID in testes of *mip40* mutants. In *mip40* background, virtually all Comr peaks disappeared: only 56 Comr peaks with *P* < 10^−3^ were detected in *mip40* mutants, while in wild-type 5422 highly specific peaks were found (see above). Comr profiles in *mip40* mutants and in wild-type are exemplified in Additional file 3: Fig. 3.

Next, we performed an analysis of the putative DNA motifs within the peaks detected for each of three proteins. Notably, Can peaks appeared to be highly enriched with the consensus sequences of Achi and Vis proteins [21] that also contribute to gene regulation in testes (Additional file 4: Fig. 4). This suggests that tTAFs may share targets with a complex containing Achi/Vis. Given the non-random coincidence of Comr and Can peaks (Additional file 1: Fig. 1), one would expect similar enrichment with DNA motifs in Comr binding sites. However, no clear consensus motifs were detected in Comr DamID peaks. Probably, Achi/Vis motif found in Can binding sites is masked by the considerable number of non-overlapping peaks between Comr and Can (Additional file 2: Fig. 2). Alternatively, different subsets of Can peaks overlap with Comr and Achi/Vis. Search in Mip40 peaks also did not yield characteristic motif, which could be explained by the involvement of Mip40 into at least two different complexes—tMAC and MMB/dREAM.

Next, we investigated the location of Can, Comr, and Mip40 peaks relative to the 1389 transcripts (Additional file 5: Table 1) that are specifically up-regulated in testes and down-regulated in other tissues (compared to the whole fly according to the FlyAtlas database, see Methods). We calculated relative occurrence of Can, Comr, and Mip40 peaks in promoters (400 bp upstream Transcription Start Sites, TSSs), 5’UTRs, exons, introns, CDSs, and 3’UTRs of these genes and found that all three factors are promoter-proximal (Fig. 2b). Statistical significance was estimated with binomial test (significance threshold *P* < 10^−3^ was applied), and expected probabilities were calculated using the genome coverage in each category. A more detailed analysis of genes having Can, Comr, or Mip40 peaks within 1 kb around their TSS demonstrated that Can preferentially binds narrowly at the TSS. Comr and Mip40 demonstrated an asymmetrical binding with the clear shift into regions upstream TSS (Additional file 6: Fig. 5). Remarkably, part of highly significant peaks localized in a considerable distance from genes (681 Can peaks, 2345 Comr peaks, and 5149 Mip40 peaks were located in the intergenic regions, at least 1 kb from the nearest TSS). This could indicate that there are long-distance regulatory effects; however, this suggestion should be tested in direct experiments.

To investigate Can, Comr, and Mip40 contribution to gene regulation, we compared gene expression in wild-type testes with that of *can, comr*, *mip40* mutant males using RNA-seq. We also generated RNA-seq data for *bam* mutant testes, as they are known to be blocked at spermatogonial stage and served as a reference point. We then explored how Can, Comr, and Mip40 are distributed around TSSs of the genes whose expression changes in the mutants. Therefore, we calculated the distance from each TSS (including alternative TSSs occurring in some genes) to the closest significant enrichment peak of these proteins. We compiled sets of genes that displayed greater than fourfold difference in gene expression and harboring Comr, Can, or Mip40 peaks within 10 kb of their TSSs. We then plotted the distribution of protein enrichment peaks in 1 kb bins around TSSs of such genes (Fig. 2c). Forty-nine percent of genes that are down-regulated at least fourfold in *can* mutants have a Can peak within 1 kb of TSS. In contrast, only 12% of genes that are up-regulated in *can* mutants have Can peaks within 1 kb of their TSSs. This difference is statistically significant as assessed by Chi-square test (*P* = 1.8 × 10^−10^). Interestingly, no such difference is observed between the genes in the next 1 kb bin (Fig. 2c). Together with the analysis in Fig. 2b, this simple test illustrates the idea that the activating function of Can is restricted to the immediate proximity of TSS of its cognate gene targets.

The same analysis applied to the Comr datasets revealed similar trend, albeit less pronounced (Chi-square test, *P* = 5.7 × 10^−5^, Fig. 2c). Somewhat surprisingly, Mip40 peaks were found to cluster around TSSs of genes that are either up- or down-regulated in *mip40* mutant testes (Fig. 2c). The fact that many Mip40-enriched genes become activated in *mip40* mutants suggests that it participates in both repressive (MMB/dREAM) and activating (tMAC) complexes.

These data allow us to determine the genes that are direct targets of the studied proteins. We strengthened the expression threshold to increase specificity: the gene was considered a direct target if it displayed at least eightfold down-regulation in the mutant and had a protein enrichment peak within 1 kb of TSS. In *comr* mutant testes, 1043 genes display greater than eightfold decrease in expression. Of these, only 232 genes have pronounced Comr peaks within 1 kb of TSS (Additional file 7: Table 2). Of 630 genes down-regulated in *can* mutants at least eightfold, only 151 genes have significant Can binding near TSS. For Mip40, we found 436 direct gene targets (Additional file 7: Table 2). The remaining genes that are affected by mutations were conditionally called indirect targets for the further analysis. It cannot be excluded that some direct target genes showing smaller expression changes or DamID values fell into the set of indirect targets. However, the whole genome analysis shows that chosen FDR-based threshold result in higher specificity of target definition (Additional file 8: Fig. 6).

Two of the three known gene targets of tTAF, *don juan*, and *Mst87F* [10] displayed pronounced Can peaks in the promoter regions (Additional file 9: Fig. 7). Thus, our data are in line with the reports [2, 10] that *dj* and *Mst87F* are directly controlled by tTAFs. Notably, though, our data imply that Comr controls these genes indirectly.

The sets of direct and indirect gene targets were very different from each other in many ways (Fig. 2D, Additional file 7: Table 2). Fifty-six percent of directly regulated Can target genes also had a Comr binding peak next to the TSS (which is 7 times over the value expected by chance, Chi-square test, *P* = 3.2 × 10^−107^). In the case of indirectly controlled gene targets, this number was only 1.44-fold above the expected value (Chi-square test, *P* = 0.004). Similarly, 78% of direct Can targets had Mip40 peaks near the TSS, which is 2.64 more frequent than expected (Chi-square test, *P* = 9.4 × 10^−17^). This is unlike the situation with indirect Can targets that appeared to associate with Mip40 at a nearly background frequency (Chi-square test, *P* = 0.11; Fig. 2d). The same overall trend was observed for Comr and Mip40 targets (Fig. 2d). This implies that more genes could be attributed to direct targets of Can, Comr, and Mip40 if milder selection criteria were applied; however, we proceeded with the gene sets described above, because they are the most prominent targets of the factors under investigation.

To summarize, Comr, Can, and Mip40 appear to directly control hundreds of genes that become activated in spermatocytes. The gene lists for direct targets display partial overlap (Additional file 10: Fig. 8). The genes that were likely to be indirect target revealed only a modest association with the Comr, Can, and Mip40 at nearly background frequencies suggesting that their activation is controlled by alternative mechanisms.

### Mutual regulation of meiosis arrest genes

In order to comprehensively analyze the mechanisms of gene activation in a complex system such as *D. melanogaster* spermatogenesis, possible cross-regulation of genes encoding tMAC and tTAFs subunits must be taken into account. Our current data (Additional file 11: Table 3) are in agreement with the previous report that Comr does not affect the activity of meiosis arrest genes [17], which led to the conclusion that tMAC is unlikely to regulate the components of tTAF.

**Fig. 3:**
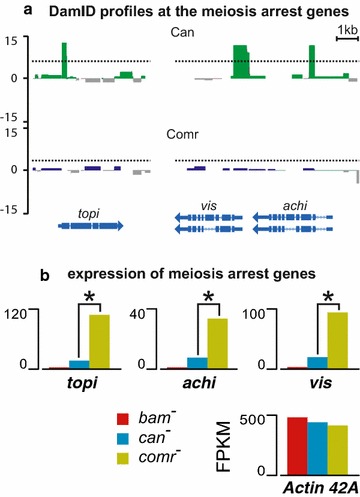
Second layer of regulation in the gene activation cascade in spermatocytes. **a** Can peaks are present in the promoters of meiosis arrest genes *topi*, *achi*, and *vis*. Dotted lines designate the values corresponding to FDR = 0.05. **b** The effects of *bam*, *can*, and *comr* mutation on expression of these genes. *Topi*, *achi*, and *vis* are inactive in spermatogonia (*bam*), yet they become activated in spermatocytes. Despite identical cell composition in the testes of *can* and *comr* mutants, the genes *topi*, *achi*, and *vis* show much weaker expression in *can* mutants (asterisk designate *P* < 0.003). As a control, the data for *Actin 42A* gene are shown to illustrate that these mutations do not influence its expression

Here, we asked whether Can, as a component of tTAF, may affect the expression of other meiosis arrest genes (Additional file 11: Table 3). Our analysis shows that three meiosis arrest genes—*topi*, *achi*, and *vis*—displayed pronounced Can binding near the TSS (Fig. 3a), suggesting that Can may be directly involved in regulation of these genes.

To check this, we looked at RNA-seq data in *can* and *comr* mutant testes. It must be noted that testes of meiosis arrest mutants accumulate spermatocytes that fail to enter downstream spermatogenesis stages. This means that some spermatocyte-specific genes may erroneously appear overexpressed in the mutant testes when matched against wild-type controls. However, we can adequately compare the expression of spermatocyte-specific genes in *can* and *comr* mutant testes, as they are composed of very similar cell types. Expression of *topi*, *achi*, and *vis* genes in *can* mutant testes was significantly reduced (multiple testing corrected *P* < 0.003 in each case) compared to *comr* mutant background (Fig. 3b), as estimated using Cuffdiff package [22] (see “Methods”). In order to independently verify this observation, we turned to the microarray data published previously [17]. As appeared, *comr* (tMAC) mutants indeed had at least tenfold higher expression of *topi*, as compared to *can* mutant animals. Notably, even in the absence of Can function, *topi* is only partially silenced compared with *bam* mutant spermatogonia (Fig. 3b). Thus, full expression of *topi* requires tTAFs activity, whereas *can* mutation significantly, yet incompletely, suppresses *topi* expression. Our data demonstrate that expression of three meiosis arrest genes depends on Can protein, suggesting that tTAFs participate in their regulation and may affect the expression of their targets.

### Activity of genes encoding TBP-like proteins in spermatogenesis

During gene activation, TAFs interact with TBP (TATA-binding protein) to form TFIID complex [23]. Similarly, tTAFs have been hypothesized to form an analogous complex, wherein the TBP-like molecule still remains to be identified [3, 9]. There are 5 genes encoding TBP and TBP-like molecules in *D. melanogaster*: *Tbp*, *Trf*, *Trf2*, *CG9879*, and *CG15398*. We analyzed the expression of these genes in the *can*, *comr*, and *mip40* mutant testes.

**Fig. 4:**
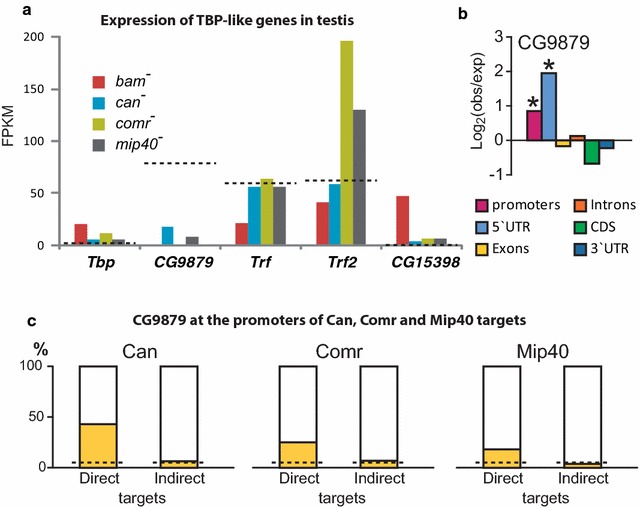
Analysis of Tbp paralogues behavior in spermatogenesis. **a** Expression of genes encoding TBP and its paralogues in the testes. *CG9879* is the only testis-specific homologue of TBP; *Trf* and *Trf2* display high expression in the testes, which remains unchanged in the mutants affecting spermatogenesis progression. *Tbp* and *CG15398* are predominantly active in spermatogonia. The dotted lines designate the FPKM expression levels of the genes in wild-type testis. **b** CG9879 displays TSS-biased binding in the set of testis-specific genes. Asterisks show the significant differences (binomial test, *P* < 0.001). **c** CG9879 is frequently found in the promoter regions of direct gene targets of Can, Comr, and Mip40, unlike in the promoters of indirect gene targets. The dotted lines show the values expected by chance

*Tbp* and *CG15398* were predominantly active in spermatogonia (in *bam* mutants) and were essentially silent in *comr*, *can*, *mip40* mutants, as well as in the wild-type background (Fig. 4a). In contrast, *Trf* and *Trf2* showed pronounced expression in all genotypes tested (Fig. 4a), which is supported by RNA *in situ* hybridization (Additional file 12: Fig. 9). *CG9879* went completely silent in *comr* mutants and showed substantial down-regulation in *can* and *mip40* mutant background (Fig. 4a) [17]. Thus, it seems probable that Trf and/or Trf2 play the role of TBP in tTAFs complex. Control of CG9879 by Comr and Can implied that it may have a specific role in gene regulation downstream tMAC and tTAFs; therefore, we performed DamID-seq for this protein and carried out RNA-seq in *CG9879* mutant testes.

The profile of CG9879 binding indicates that this protein tends to associate with 5’UTR and promoter regions of the genes that are specifically activated in testis (binomial test, *P* < 0.001, Fig. 4b, Additional file 13: Fig. 10). Using DREME platform, we found that CG9879-bound regions frequently contained AT-rich motifs resembling the TATA-box sequence (Additional file 14: Fig. 11) [24]. In general, CG9879 tends to co-localize with both Comr and Can (Additional file 1: Fig. 1). Furthermore, 43% of direct Can gene targets had a CG9879 peak within 1 kb around TSS (8.4-fold above expected, Chi-square test, *P* = 5.2 × 10^−14^). Direct gene targets of Comr and Mip40 had peaks of CG9879 near TSSs in 25% (fivefold enrichment, Chi-square test, *P* = 2.0 × 10^−44^) and 18% (3.6-fold enrichment, Chi-square test, *P* = 1.9 × 10^−9^) cases, respectively (Fig. 4c). Notably, genes that we considered to be indirectly regulated by Comr, Can, and Mip40 were not enriched with CG9879 peaks (Fig. 4c, Additional file 7: Table 2).

In order to understand how CG9879 affects gene expression in fly testes, we knocked out *CG9879* using CRISPR/Cas9 (see “Methods”). Surprisingly, no morphological defects were apparent, and the males remained fully fertile. Furthermore, analysis of gene expression in testes of *CG9879* mutants showed that only 28 genes had significantly reduced expression levels (Additional file 15: Table 4), but none of them was associated with CG9879. Taking into account our data on specific binding of CG9879 to direct tTAFs and tMAC targets, this lack of phenotype and expression changes is likely attributable to the redundancy of CG9879 in the presence of other TBPs (Trf, Trf2) that may completely substitute its function.

### tMAC is required for Mip40 recruitment to the promoters of testis-specific genes

One intriguing feature of spermatocyte-specific gene activation program is participation of Mip40 (Fig. 1b). Mip40 protein was identified as the subunit of MMB/dREAM complex that is present in various cell types [12–14, 25–27]. Mip40 is also an essential component of tMAC [4].

Given an extensive similarity between the components of tMAC and MMB/dREAM complexes (Fig. 1b), it is possible that in spermatogonia MMB/dREAM complex is bound to the spermatocyte-specific genes thereby keeping them silent. Upon spermatocyte differentiation, the components of tMAC could replace homologous proteins in the MMB/dREAM and turn it into a transcriptional activator. On the other hand, tMAC could recruit the components of MMB/dREAM to the spermatocyte-specific genes resulting in tMAC-dependent recruitment of Mip40 following spermatocyte differentiation.

**Fig. 5:**
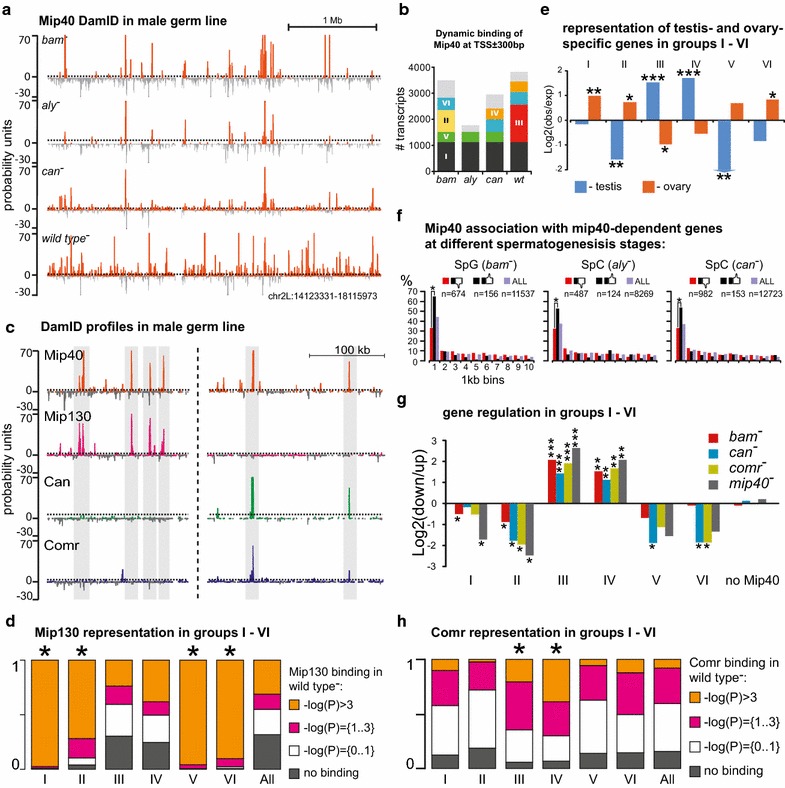
Mip40 shows highly dynamic chromatin binding during spermatogenesis. **a** Mip40 binding profiles in spermatogonia (*bam* mutants), in *aly* mutant testes (no tMAC), *can* mutant testes (no tTAFs), as well as in the wild-type testes. **b** Basic types of changes in Mip40 binding to the genes. The genes having Mip40 peaks within 300 bp around TSS were identified in each genotype and sub-classified to six groups (I–VI) reflecting the main trends of Mip40 binding across the genotypes tested: group I was associated with Mip40 in all four genotypes, group II was bound by Mi40 only in spermatogonia of *bam* mutants, and so on (see the main text for details). **c** Mip130 coincides with Mip40 binding sites devoid of Can or Comr and is absent from the sites of Mip40 colocalization with Comr or Can. **d** Mip130 occupancy in the gene groups showing dynamic binding of Mip40 (Fig. 5B). Mip130 DamID signal was categorized by the significance of DamID signal: no Mip130 binding; − log(*P*) = {0..1}—nonsignificant binding; − log(*P*) = {1..3}—medium significance peaks; − log(*P*) > 3—highly significant Mip130 peaks. Mip130 strongly marks the genes in groups I (Chi-square test, *P *< 10^−300^), II (Chi-square test, *P *= 1.8 × 10^−155^), V (Chi-square test, *P *= 5.9 × 10^−161^), and VI (Chi-square test, *P* = 3.4 × 10^−163^). Groups III and VI show Mip130 presence similar to random expectation. **e** Enrichment or depletion in testis-specific genes (blue) and ovary-specific genes (orange) in the six gene sets (***P < 10^−31^; **P < 10^−6^; *P < 10^−2^, Chi-square test). **f** Distribution of Mip40 around the transcripts showing greater than fourfold difference in gene expression (in *mip40* vs. wild-type testes) according to DamID in *bam*, *aly*, and *can* mutants. For each transcript, the distance between its TSS and the closest Mip40 peak was calculated and plotted in 1 kb bins within 10 kb around the TSS. Asterisks indicate that the differences among the groups of transcripts showing greater than fourfold changes in expression are statistically significant. Binding of Mip40 in spermatogonia, as well as in the absence of tTAFs and tMAC, is predominantly associated with gene repression. **g** Ratios of repressed and activated genes in the groups of genes shown in Fig. 5b. Mip40-bound gene targets in spermatogonia tend to be up-regulated in *mip40* mutants (groups I, II, V, and VI). The genes that acquire Mip40 binding following spermatocyte differentiation via tMAC activity (groups III and IV) show overall decreased expression in *mip40* mutants, i.e., Mip40 in this case is needed for their activation (***P < 10^−30^; **P < 10^−10^; *P < 10^−3^, Chi-square test). **h** Presence of Comr protein in the promoters of the genes from the six groups of genes in Fig. 5b. Comr binding in wild-type testes was categorized by the significance of DamID signal: no Comr binding; − log(*P*) = {0..1}—nonsignificant binding; − log(*P*) = {1..3}—medium significance peaks; − log(*P*) > 3—highly significant Comr peaks. High and medium significance peaks of Comr are overrepresented in the groups III (Chi-square test, *P* = 1.3 × 10^−96^) and IV (Chi-square test, *P* = 1.2 × 10^−110^)

In order to understand which of the scenarios operates leading to activation of spermatocyte-specific genes, we performed DamID profiling of Mip40 in testes from *bam*, *aly*, and *can* mutants and compared these profiles to each other and to the profile from wild-type testes. In spermatogonia of *bam* mutants, where tMAC is not yet expressed, Mip40 profile represents MMB/dREAM complex (Fig. 5a). In *aly* mutants, many Mip40 peaks were absent and some novel Mip40 sites appeared. In contrast, novel Mip40 binding sites—absent in both spermatogonia and *aly* mutants—were readily detectable in *can* mutant background (Additional file 16: Fig. 12). In wild-type testes, these novel peaks were even more prominent, and the profile was very different from that of the spermatogonial cells (Fig. 5a).

In order to analyze these effects in relation to gene regulation, we focused on the transcripts having Mip40 peaks within ± 300 bp of the TSSs in each genotype. Overall, there were half as many Mip40-occupied TSSs in *aly* mutants (1773 genes) compared to *bam* mutants (3499 genes) (Fig. 5b). In contrast, in *can* mutant and wild-type testes, the numbers of Mip40-positive TSSs increased (2950 and 3819 genes, respectively). To reveal the main trends in Mip40 profile dynamics, we performed clustering of these genes depending on how they associate with Mip40 during spermatogenesis and six major gene groups were formed (Fig. 5b). These six groups are reproducible across different significance levels chosen for Mip40 peak calling (Additional file 17: Fig. 13).

Since Mip40 is shared by MMB/dREAM and tMAC, its DamID profile likely represents a superposition of two profiles. To distinguish between tMAC and MMB/dREAM localization, we generated an additional DamID profile of specific subunit of MMB/dREAM Mip130, which is homologous to Aly protein but does not participate in tMAC (Fig. 1b), and compared it with the profiles of Mip40 as well as Comr and Can. Mip130 proved to co-localize with Mip40 at numerous genomic locations (Fig. 5c). Characteristically, these have virtually no overlap with the sites of Comr and likely represent the MMB/dREAM localization (Additional file 18: Fig. 14). On the other hand, the sites of Mip40 that coincide with Comr do not typically contain Mip130, thus reflecting tMAC position (Fig. 5c). Accordingly, Mip130 revealed differential representation in 6 gene groups that reflect the main trends of Mip40 redistribution (Fig. 5d), allowing to discriminate Mip40 as a part of tMAC or MMB/dREAM. A highly specific enrichment with Mip130 was observed in groups I, II, V, and VI in comparison with the genome-wide overall distribution (Chi-square test, *P* < 10^−154^, Fig. 5d): cooperative signal of Mip40 and Mip130 in these groups indicates the MMB/dREAM binding. The groups III and IV demonstrated no prevalent Mip130 presence suggesting that Mip40 signal in these groups is due to tMAC formation (Fig. 5d).

We used 2252 shared peaks of Mip40 and Mip130 to characterize sequence motifs in MMB/dREAM sites (Additional file 19: Fig. 15). The best motif identified in this search manifested high similarity with the motif for BEAF-32 protein, which is known to interact with CP190 protein at the insulator sites [28]. In turn, CP190 was found to interact with MMB/dREAM complex [29]. Thus, the presence of BEAF-32 motif in the Mip40 and Mip130 binding sites could reflect the similar involvement of MMB/dREAM in regulation of promoter-enhancer regulation in germline.

To check whether the observed dynamics of Mip40 profile is specific for genes involved in spermatogenesis, we turned to the set of 1389 testis-specifically expressed genes (see above). As a control, we generated a list of 707 ovary-specifically expressed transcripts (Additional file 5: Table 1) selected with the same criteria from the FlyAtlas database (Methods). In the groups of genes that display Mip40 binding at the spermatogonial stage (groups I, II V, VI), testis-specific genes were underrepresented, whereas the fraction of ovary-specific genes was above the expected value (Fig. 5e). Among the genes whose TSSs acquire Mip40 binding in spermatocytes and onwards (groups III and IV) testis-specific genes were highly overrepresented (Fig. 5e). Thus, upon spermatocytes differentiation, Mip40 relocates to the promoters of testis-specifically expressed genes in tMAC-dependent manner.

We showed that *mip40* mutation results in down-regulation of 1580 transcripts (at least fourfold in *mip40* mutant testes vs. wild-type controls) but also in fourfold up-regulation of 208 transcripts (Fig. 2c). These effects are probably caused by participation of Mip40 in two types of complexes, one of which would cause gene repression (like MMB/dREAM), while the other being an activator (tMAC). To investigate the repressive effects of Mip40, we checked how this protein is associated with those two gene sets throughout the first stages of spermatogenesis: in spermatogonia of *bam* mutants and in spermatocytes of *aly* and *can* mutants. Therefore, we analyzed Mip40 binding within 10 kb of the TSSs of transcripts from these two sets. In spermatogonia, 60% of transcripts that are up-regulated in *mip40* testes had a Mip40 peak within 1 kb of the TSS (Fig. 5f). These transcripts are normally repressed, and Mip40 is associated with them already in spermatogonia. In contrast, only 30% of the fourfold down-regulated genes contained Mip40 near their TSS, which corresponds to the random expectation and is significantly less than the portion of up-regulated genes (Chi-square test, *P* = 5.5 × 10^−13^, Fig. 5f). Similar yet less pronounced situation was observed in spermatocytes of *aly* and *can* mutants (Chi-square test, *P* = 4.8 × 10^−5^ and *P* = 1.2 × 10^−6^, respectively, Fig. 5f). These data indicate that in spermatogonia and in early spermatocytes of *aly* and *can* mutants Mip40 directly binds to a large portion of genes that should be down-regulated in spermatogenesis.

To check this further, we generated the fly strain bearing both *mip40* and *bam* mutations. This strain allowed us to estimate the effect of Mip40 on gene expression selectively in the spermatogonia. Analysis of expression in *mip40*; *bam* double mutant testes revealed that the genes that are up-regulated in this genotype relative to *bam* mutants tend to bind Mip40 in spermatogonia. This means that the presence of Mip40 at their promoters correlates with their repression (Additional file 20: Fig. 16). Notably, later in development, neither Can nor Comr showed significant association with the same gene sets indicating that tMAC and tTAFs play no role in their regulation (Additional file 20: Fig. 16).

Figure 5b indicates that tMAC and tTAFs affect Mip40 binding in distinct gene groups. In order to estimate how this is related to gene regulation in the six major groups shown in Fig. 5b, we turned to our differential gene expression datasets for *bam*, *comr*, *can*, and *mip40* mutant testes. In each mutant background, the transcripts that showed at least fourfold up- or down-regulation relative to the wild-type control were retained. Next, we calculated the ratio of repressed to activated transcripts in the groups I–VI (Fig. 5g).

In the group I transcripts (bound by Mip40 throughout all stages and genetic backgrounds), a strong enrichment for transcripts up-regulated in *mip40* mutants was observed (in comparison with expected value), and so Mip40 likely acts as a repressor for such genes (Fig. 5g). This effect was specific to *mip40* mutants, as it was not observed in *can* and *comr* mutants, which in turn indicates that tMAC and tTAFs do not significantly affect the regulation of group I transcripts. The transcripts from groups II, V, and VI (whose TSSs show Mip40 binding in spermatogonia) likewise show enrichment for genes up-regulated in *mip40* mutants. However, unlike in group I, these genes were also up-regulated in *can* and *comr* mutant backgrounds (Fig. 5g). Nonetheless, only a handful of TSSs from these groups are directly bound by Comr or Can (data not shown), and so tMAC and tTAFs are inferred to have indirect effects on expression of these genes. One could suggest that the genes from the groups II, V, and VI that are repressed by Mip40 in spermatogonia are activated upon spermatocyte differentiation independently from tMAC and tTAF. In this case, their up-regulation in *can* and *comr* mutants would be explained by spermatocyte accumulation.

In contrast, the transcripts, whose TSSs for the first time recruit Mip40 in spermatocytes (groups III and IV), tend to show reduced expression in *mip40* mutants, which argues for the activating role of Mip40 for group III and IV genes (Fig. 5g). Notably, these genes also tend to be Comr targets: 64% transcripts co-bound by Mip40 and Comr in wild-type belong to the groups III and IV. In other words, such transcripts appear to be directly activated by Mip40 and Comr in the context of tMAC (Fig. 5h).

Thus, our data indicate that in spermatogonia Mip40 plays a repressive role. Following spermatocyte differentiation, relocalization of Mip40 occurs, and tMAC but not tTAFs components are required for this relocalization. Establishing the final Mip40 distribution pattern is only possible when both complexes are available. The Mip40 redistribution to the promoters of testis-specific genes is indispensable for their proper activation.

## Discussion

The present work aims at extending our knowledge of the mechanisms of massive gene activation controlled by tMAC and tTAFs complexes in Drosophila spermatocytes. We performed comprehensive genome-wide analyses that uncovered new trends in this process.

### DamID data criticism

Before proceeding to the discussion of the intricate biological effects observed, it is important to address the question of whether DamID system accurately represents the dynamic events of transcription factor binding in fly testes. Indeed, in our DamID experiments the removal of transcription terminator stuffer otherwise blocking transcription of Dam-fusion protein is mediated by CRE that is produced early in the stem cells of the germline [16, 17]. Hence, Dam-mediated methylation of DNA may occur at any of the subsequent developmental stages—in spermatogonia, spermatocytes, and spermatids—that all can contribute to the ultimate binding profile. Accordingly, changes in the ratios of cell types between the genotypes may be a confounding factor. On the other hand, in wild-type testes, as well as in meiotic arrest mutants, the fraction of spermatogonial cells among all cell types of the testis is very small and should have little if any influence on the profiles obtained.

Our data may help to address this concern. For example, Mip40 protein was mapped in *bam* mutant testes at TSSs of nearly 3500 genes (Fig. 5b). Should the contribution of spermatogonial cells into Mip40 binding profiles in *aly*, *can*, and wild-type backgrounds be significant, Mip40 peaks observed in spermatogonia should also be present in such samples, likely having reduced magnitude. This was not the case, as in *aly* mutants roughly half the peaks disappear from the promoter regions, whereas the other half of the peaks remains unchanged (Fig. 5b). Moreover in *can* mutants and in wild-type testes, many more Mip40 peaks appear and these map to the Mip40-negative genomic loci in spermatogonial cells (Fig. 5a, b). This acquisition of novel Mip40 sites is consistent with continued DamID activity in spermatocytes. Thus, the approach used in our study can be applied for chromatin profiling in spermatogenesis and the data obtained faithfully reproduce protein binding dynamics in the dominant cell populations in each of the genotypes tested.

### Activation of spermatocyte-specifically expressed genes

The process of gene activation now appears to be somewhat different from earlier models. First, only fraction of spermatocyte-specific genes undergoes direct tTAFs- or tMAC-mediated activation. Second, regulatory cascades downstream of tMAC and tTAFs may involve other transcription factors, including those that are not particularly testis-specific. For instance, there are many transcription factors, such as *invected*, *apontic*, *fushi tarazu*, *gooseberry*-*neuro*, whose expression pattern is detected in, but not restricted to, testes [17]. Thus, the role of tMAC and tTAFs may be to launch the testis-specific gene program that unfolds via other regulators and transcription factors that ultimately results in appropriate gene activation.

It is interesting to note that tTAFs actually control expression of several meiosis arrest genes, *topi*, *achi*, and *vis*. Achi and Vis proteins are absent from the canonical tMAC complex, yet they were found in the context of a distinct complex encompassing Aly and Comr [4, 5]. In *can* mutant background, *topi*, *achi*, and *vis* undergo only partial down-regulation, and so this may explain why *can* mutation has a weaker phenotype compared to that of *topi/achi/vis* knock-outs, although this may also be interpreted the other way around, namely that reduced expression of these genes is partially responsible for the *can* phenotype.

It is highly probable that tTAFs forms a transcription factor paralogous to TFIID [3, 9, 10]; however, TBP protein that forms the core of tTAFs complex was not identified. An attractive hypothesis that the spermatocyte-specifically expressed TBP-like protein CG9879 may play the central role in tTAFs function was rejected in our study. Indeed, knock-down of *CG9879* gene led to very subtle changes in gene expression and did not appreciably affect spermatogenesis. Nevertheless, CG9879 tends to co-localize with tTAFs subunit Cannonball implying that CG9879 participates in tTAF, but its absence may be compensated by other TBP-like proteins expressed in spermatocytes. Such redundancy may help to maintain the stability of this important genetic system.

### Dual role of Mip40

Since the description of tMAC, one of the most intriguing facets of this complex was the homology of its subunits to those of MMB/dREAM complex. tMAC and MMB/dREAM complexes are not merely paralogous, and they share common subunits, Mip40 and CAF1-55. Notably, tMAC is clearly involved in gene activation [3, 17, 30], whereas MMB/dREAM predominantly has repressive activity [12–14], although several examples showing its activating effects have also been reported [25–27].

Our data indicate that at these early differentiation stages, Mip40 does not tend to associate with TSSs of genes that will later become activated in spermatocytes. This observation is in obvious conflict with the idea that MMB/dREAM orchestrates the repression of spermatocyte-specific genes in undifferentiated cells. Moreover, Mip40-bound genes in spermatogonia are those whose expression is predominantly detected in ovaries, and Mip40 binding in the context of MMB/dREAM complex has inhibitory activity. Whether this mechanism is related to the recently discovered pathway that maintains the silencing of somatically expressed genes [15] remains to be discovered.

Following spermatocyte differentiation, Mip40 binding pattern changes substantially, and novel Mip40 peaks appear that are clearly tMAC dependent. These data indicate that MMB/dREAM does not contribute to inactivation of spermatocyte-specific genes. Instead, in spermatocytes, tMAC recruits Mip40 to novel binding sites and this redistribution takes place outside the context of MMB/dREAM complex.

In wild-type testes, redistribution of Mip40 is much more pronounced. This points to the possible involvement of tTAFs. Alternatively, in early spermatocytes of *can* mutants we may actually observe very first steps of Mip40 redistribution, whereas more differentiated cell types are present in wild-type testes and so they may contribute to the final binding pattern. A test to discriminate between the two possibilities is to perform DamID in *thoc5* mutants, as this mutation does not interfere with tMAC or tTAFs activity, yet it causes meiotic arrest [31].

## Conclusions

Based on our major findings, we propose an amended picture of transcription-related events during Drosophila spermatogenesis. The mechanism controlling the inactivity of the vast majority of spermatocyte-specific genes is presently unknown: a decisive role for either the Pc [11] or MMB/dREAM complexes now seems unlikely. tMAC and tTAFs associate with their cognate gene targets and induce their activation. Surprisingly, of all the testis-specific genes, the fraction of high confidence direct gene targets of tMAC and tTAFs is relatively modest. Activation of indirectly controlled gene targets likely proceeds with the help of other transcription factors. Involvement of tTAFs in regulation of three meiosis arrest genes should be taken into account as an additional regulatory mechanism. There is a major redistribution of Mip40 in spermatocytes. This process is tMAC dependent and leads to the relocation of Mip40 to promoters of spermatocyte-specific genes leading to their activation.

## Methods

### Genetic constructs

All genetic constructs for DamID experiments were based on the hsp70 > loxP-Stop-loxP > Dam (JN993988) vector encompassing a loxP-flanked stop-cassette placed between the *hsp70* minimal promoter and the Dam CDS, fused in frame as an N-terminal fusion to the protein of interest [32]. The Dam-Comr (KC845569) construct has been reported earlier [17]. Dam-Can (KY939771), Dam-Mip40 (KY939772), Dam-Mip130 (MG557560), and Dam-CG9879 (KY930504) constructs were generated in this work.

### Fly stocks and crosses

To obtain fly stocks needed for DamID experiments, attP40 genomic landing site on chromosome 2 was used (Dam-Comr, Dam-Mip40, Dam-Can, Dam-CG9879, and Dam-alone). To activate the DamID system specifically in the male germline, *nanos*-*cre* (attP40) males [16, 17] were crossed to DamID-construct bearing females. In the progeny of these crosses, removal of the stop-cassette occurs only in the germline cells, but not in the somatic cells of the testis. Dam-alone flies were used as a control for DamID experiments.

To perform DamID in animals displaying compromised spermatogonia-to-spermatocyte differentiation (*bam*-*delta86)*, tMAC activity (*aly*^*5*^) or tTAFs activity (*can*^*1*^), flystocks having said mutations balanced against TM6 and homozygous for Dam-Mip40 (attP40), Dam-alone (attP40) or *nanos*-*cre* (attP40) constructs were established by standard genetic crosses. When DamID; mut/TM6 females were crossed to nanos-cre; mut/TM6 females, their sons lacking TM6-linked dominant markers and therefore homozygous mutant were selected. Such males displayed the expected phenotypes: accumulation of spermatogonia (*bam*) or spermatocyte meiotic arrest (*aly* and *can*). Comr profiling at the *mip40*^*EY16520*^ background was performed using the same experimental design.

### Germline-specific DamID

For DamID experiments, testes were collected from 3-day-old males. For each biological replicate, 50 pairs of testes were used; each experiment was performed in two biological replicates. Standard phenol-chloroform extraction method was used to isolate genomic DNA from the collected material. 0.5–1 μg DNA was used in each DamID experiment. Overall, DamID was performed according to the protocols published previously [33, 34] with modifications [18]. Specifically, the last amplification step was done using regular Taq-polymerase. Following amplification, the PCR products were treated with DpnII to remove adapter sequences. Next, library preparation followed the TruSeq protocol (Illumina) omitting the additional fragmentation step. Importantly, this helps retain the information on the sequences that must be found at the amplified DNA termini, as they must begin with GATC. This information is used for downstream data filtering and removal of non-specific reads as previously described [18]. Further analysis, including profiles generation and peak calling, was performed exactly as previously described [18]. In all cases, FDR cutoff was required to be 0.05 at most. FDR estimation was performed at different significance levels to assess the impact of experimental noise measured by comparison of biological replicates. Additional file 21: Fig. 17 exemplifies the outcome of this procedure on Can DamID-seq data. Additional file 22: Fig. 18 illustrates the benefits of this approach as compared to traditional DamID data presentation as log_2_(Dam-*X*/Dam) on the same dataset.

### Gene expression analysis

For gene expression analysis, we used 50 adult testes from 3-day-old wild-type males (*y*^*1*^,*w*^*67*^ strain) or homozygous mutants for *bam*^*delta86*^, *aly*^*5*^, *can*^*1*^, *mip40*^*EY16520*^ or *CG9879* (obtained in this study). Each experiment was run in duplicate. Total RNA was isolated from testes, using TRIZol (Invitrogen) reagent, according to the manufacturer’s instructions. One microgram of total RNA was then processed for library preparation using the RNA TruSeq kit. The libraries were sequenced on the Illumina MiSeq system (paired reads, 75 bp). Data were analyzed using Galaxy tools: reads were aligned on *D. melanogaster* BDGP R5/dm3 genome assembly (https://genome-euro.ucsc.edu/) using TopHat (− r 200 − mate-std-dev 50) [35]. Transcript differential expression testing between samples was performed with Cuffdiff using geometric normalization, pooled dispersion estimation, and FDR = 0.05 [22].

### Testis-specific and ovary-specific transcripts

To determine the list of testis- and ovary-specific transcripts, we used FlyAtlas Database [1]. We used following criteria to assign transcript as a testis-specific (or ovary-specific)—it should be up-regulated in testis (or ovary) (log_2_(Testis(Ovary)/FlyMean) > 0) and down-regulated (log_2_(Tissue/FlyMean) < 0) or demonstrate null expression in all other tissues of adult fly. This approach allowed us to generate the list of 1389 testis-specific and 707 ovary-specific transcripts.

### CRISPR/Cas9 genome editing

To generate full-size deletion of *CG9879* gene coding sequence, we used transgenic line MI04214 from MiMIC transposon insertion collection (Bloomington Drosophila Stock Center, [36]). This stock contains insertion of MiMIC transposon carrying a marker gene (*y*^+^) in approximately 600 bp from 5′ end of *CG9879*. MI04214 flies were crossed to the flies bearing Cas9 nuclease gene (#51326, Bloomington Drosophila Stock Center) (Additional file 23: Fig. 19). Oligonucleotides that target the genomic region that contains MiMIC insert and CDS of *CG9879* were designed with CRISPR optimal finder and CRISPRdirect tools [37, 38]. Each oligonucleotide pair (L1: 5′-cttcgacgatggtgacaggtgtct-3′, L2: 5′-aaacagacacctgtcaccatcgtc-3′, R1: 5′-cttcgtgccagtggttggcccgag-3′, R2: 5′-aaacctcgggccaaccactggcac-3′) were annealed on each other and inserted into pU6-BbsI-chiRNA vector (Addgene, #45946, [39]). Plasmids encoding a chiRNA targeting the genomic region of interest were co-injected into preblastoderm embryos obtained from the crosses mentioned above. Upon eclosion, flies were crossed to *y, w* flies, and progeny of these crosses were inspected for loss of yellow dominant marker that expected to occur in the case of successful deletion of MiMIC insert and coding region of *CG9879*. As a result, we obtained flies, bearing required deletion (Additional file 23: Fig. 19). Complete deletion of *CG9879* coding region was verified with PCR and Sanger sequencing (Additional file 23: Fig. 19).

## Additional files

**Additional file 1: Fig. 1.** Can, Mip40, Comr, and CG9879 tend to co-localize in the genome. **A** Can peaks (top plot) and Comr peaks (bottom plot) were centered. The plots show the ratio of observed/expected frequencies of other protein factors as a function of distance from the centers of Can and Comr peaks. In Can peaks, Comr and CG9879 are 17- and 18-fold more frequent than expected by chance. Mip40 protein shows sevenfold enrichment. Random set of GATC sequences is shown in purple. The situation for Comr peaks is slightly different: Can, Mip40, and CG9879 proteins show 15-, 8-, and ninefold enrichment at these regions above expected by chance. The observed enrichment quickly drops at increasing distances from the centers of the peaks. Observed enrichments over the expected value were highly significant for all proteins as assessed by the binomial test, with the highest significance at zero position (*P* < 10^−212^ in centered Can peaks and *P* < 10^−144^ in centered Comr peaks). **B** Examples of testis-specifically expressed genes containing Can, Comr, and Mip40 peaks in the vicinity of TSS. Ordinate is the same as in Fig. 2a.

**Additional file 2: Fig. 2** Coincidence of peaks of Can, Comr, and Mip40. Euler diagram represents the exact intersections of the peaks detected for Can, Comr, and Mip40 proteins in wild-type testes. The numbers of peaks in each partition are shown.

**Additional file 3: Fig. 3.** DamID of Comr in *mip40* mutants. Comr is unable to bind the chromosomes in absence of Mip40 (upper profile). Comr profile in wild-type is presented for comparison (lower profile).

**Additional file 4: Fig. 4.** Motif analysis in Can peaks. Top three motifs found by DREME [24] in Can binding sites are shown. Below is the analysis of the top-most motif identified by TomTom software [40]. This motif perfectly matches the motifs of Achi and Vis.

**Additional file 5: Table 1.** The lists of testis- and ovary-specifically expressed genes used in this study.

**Additional file 6: Fig. 5.** Averaged DamID profiles of Can, Comr, and Mip40 at the TSS of genes. The genes having the peaks of Can, Comr, and Mip40 within 1 kb around their TSS were selected. Then, for each coordinate within this area the normalized number of peaks was calculated. Ordinate–relative abundance of each protein. Black line represents the randomized set of peaks.

**Additional file 7: Table 2.** Direct and indirect gene targets of the proteins studied. Direct gene targets are defined as the genes that have a significant peak of a given protein within 1 kb of their TSSs and showing at least eightfold down-regulation in the corresponding mutant. Genes showing reduced expression but lacking detectable protein factor binding within such distance are referred to as indirect targets. Primary data on protein binding and expression are shown in the worksheets.

**Additional file 8: Fig. 6.** DamID signal at the TSSs of genes regulated by Comr, Can, and Mip40. Using RNA-seq data, the genes were determined that demonstrate at least fourfold expression change in *comr*, *can*, or *mip40* mutant testes. DamID signal of the corresponding factor was classified at ± 1 kb around TSSs of these genes. The categories were as follows. No binding shown in gray (DamID signal of the protein is lower than of Dam-alone control: Dam-*X*/Dam < 1, where *X* = Comr, Can or Mip40). The genes that manifest only insignificant DamID signal (− log(*P*) = {0..1}, where *P* is a Fisher’s exact test *P* value) are shown in white. The genes with moderate significance level of binding are shown in purple (−log(*P*) = {0..*X*}, where *X* is a threshold determined for FDR = 0.05. Orange shows the highly significantly bound genes that show DamID signal above the FDR = 0.05 threshold.

**Additional file 9: Fig. 7.** Binding of Can, Comr, and Mip40 to model genes referenced in earlier reports [2, 10]. *Dj* and *Mst87F* genes have prominent Can peaks (DamID) in their promoter regions, which is consistent with the published ChIP-qPCR data. Comr is not associated with these gene promoters, as assayed by DamID. Mip40 peak maps to the *Mst87F* promoter.

**Additional file 10: Fig. 8.** Overlap between direct gene targets of Comr, Can, and Mip40.

**Additional file 11: Table 3.** Interplay between meiosis arrest genes. tMAC does not affect the expression of genes encoding tTAFs. Can mutation results in reduced expression of *topi*, *achi*, and *vis*. Additionally, Can peaks are detectable in the immediate vicinity of TSSs of these genes.

**Additional file 12: Fig. 9.** RNA *in situ* hybridization localization of *CG9879*, *Trf*, and *Trf2* transcripts. Upper row—in wild-type testis; lower row—in meiosis arrest mutants. *CG9879* transcript is not expressed in *aly* mutants, while *Trf* and *Trf2* retain their expression in mutant spermatocytes.

**Additional file 13: Fig. 10.** Averaged DamID profile of CG9879 at the TSS of genes. The genes having the peaks of CG9879 within 1 kb around their TSS were selected. Then, for each coordinate within this area the normalized number of peaks was calculated. Ordinate–relative abundance of each protein. Red line represents the randomized set of peaks.

**Additional file 14: Fig. 11.** Analysis of short motifs in the CG9879 peaks. Top three most frequent motifs found in CG9879 peaks using DREME [24] are shown. AT-rich motifs are similar to TATA-box sequences.

**Additional file 15: Table 4.** The list of 28 genes that display substantially reduced expression in testes of *CG9879*-null males.

**Additional file 16: Fig. 12.** Overlap of Mip40 peak sets in *bam*, *aly*, and *can* mutants. Euler diagram represents the exact intersections of the peaks detected for Mip40 protein in three genotypes. The numbers of peaks in each partition are shown.

**Additional file 17: Fig. 13.** The dynamics of Mip40 binding during spermatogenesis, as assayed at varying levels of significance levels for Mip40 peak calling. The same groups of target genes are identified, as those shown in Fig. 5b. **A** The threshold for peak calling *P* > 10^−3^. **B** The threshold for peak calling *P* > 10^−6.25^.

**Additional file 18: Fig. 14.** Coincidence of peaks of Mip130, Comr, and Mip40. Euler diagram represents the exact intersections of the peaks detected for Mip130, Comr, and Mip40 proteins in wild-type testes. The numbers of peaks in each partition are shown.

**Additional file 19: Fig. 15.** Motif analysis in shared peaks of Mip40 and Mip130. Top three motifs found by DREME [24] in shared peaks of Mip40 and Mip130 are shown. Below is the analysis of the top-most motif identified by Tomtom software [40]. This motif perfectly matches the motif of BEAF-32 protein.

**Additional file 20: Fig. 16.** Effect of *mip40* mutation on gene expression in spermatogonia. Expression in *mip40*; *bam* double mutants was compared to *bam* mutants. Up- and down-regulated genes were determined and matched to Mip40 DamID profile in *bam* mutants. Ordinate: percent of genes in each group having one of transcription factors peaks (TF = Mip40, Comr or Can) in promoter in genotypes indicated on the abscissa. The genes that are up-regulated in *mip40*; *bam* double mutants are significantly more frequent targets of Mip40 in spermatogonia. The same genes are not enriched with Can or Comr DamID signals at the later cell stages.

**Additional file 21: Fig. 17.** Performance of peak calling algorithm illustrated by Can DamID data. **A** Numbers of Can peaks and false positive peaks at different significance levels. FDR=0.05 is reached at the *P* = 1.7 × 10^−7^ (dashed line). **B** Specificity of peak calling at different FDR values. At high FDR values, there is no prevalence of Can to the genes that are down-regulated in can mutants over the genes that are up-regulated. At FDR = 0.05, the number of down-regulated genes is about fourfold higher than the number of up-regulated genes.

**Additional file 22: Fig. 18.** Example of Can DamID data. Red profile—raw reads from Dam-Can sample. Blue profile—raw reads from Dam-alone sample. These two profiles were normalized and presented as log_2_(Dam-Can/Dam) values or treated by the previously published algorithm and shown in − log(*P*) units where *P* is the significance value (*P* value) measured using Fisher’s exact test [18]. Gray shading demonstrates the DNA fragments that demonstrate considerable Dam-Can:Dam ratio but are statistically insignificant. These fragments show low values in both Dam-Can and Dam-alone channels (arrowheads). In contrast, the peaks determined by our algorithm [18] demonstrate a high prevalence of Dam-Can signal and are located in the promoters of genes that are highly expressed in testis (shown below in red). Two genes that are marked with asterisks are predominantly expressed in spermatogonia and are not regulated by Can. The frame shows the hotspot of Dam methylation in both samples.

**Additional file 23: Fig. 19.** Targeted deletion of *CG9879* gene using CRISPR/Cas9. **A** The strain with the insertion of transposon containing *yellow* reporter gene 600 bp upstream *CG9879* gene was used. chiRNAs were designed flanking the transposon and *CG9879* gene. Targeted sequences are shown. Cas9 introduced the double-strand breaks within the targeted sequences as shown. Upon repair, the fragment containing transposon with the yellow reporter and *CG9879* gene was deleted and the new junction appeared. Deletion was confirmed by Sanger sequencing (**B**).

## Abbreviations

tMAC: testis-specific meiosis arrest complex
tTAF: testis-specific TBP-associated factor
TBP: TATA-binding protein
MMB: Myb-MuvB complex
dREAM: DP, RB-like, E2F4, and MuvB complex
DamID: DNA-adenine methyltransferase identification
