# Supplementary figures and images for: Genome-wide analysis of gene regulation mechanisms during Drosophila spermatogenesis
Source: Epigenetics Chromatin. 2018 Apr 2;11:14. doi: 10.1186/s13072-018-0183-3 (PMC5879934; doi:10.1186/s13072-018-0183-3)

*CG9879*

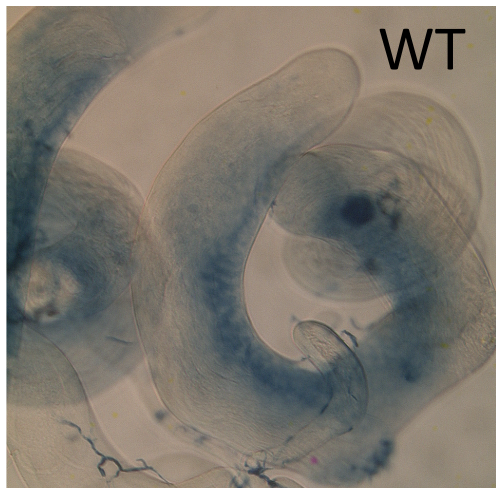

*TRF2*

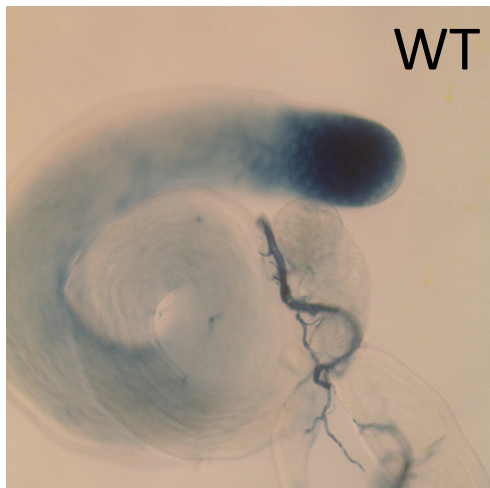

*TRF*

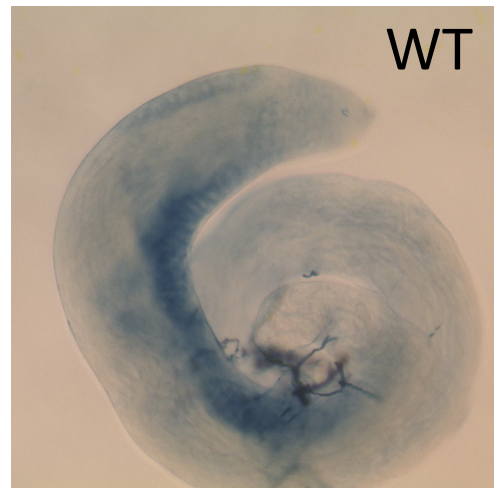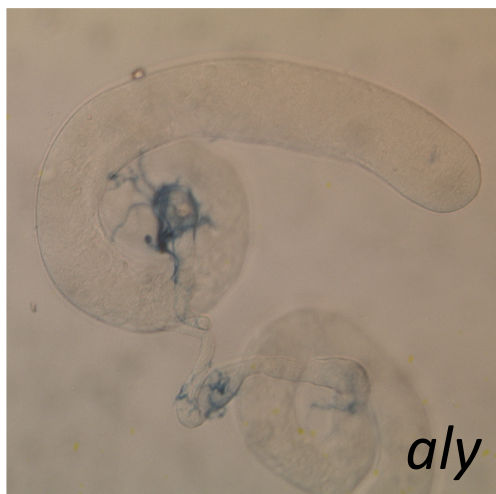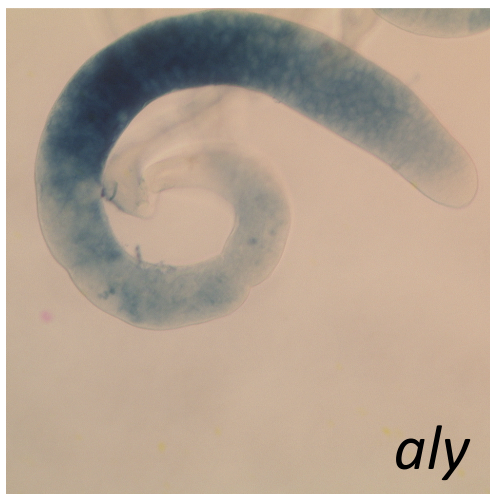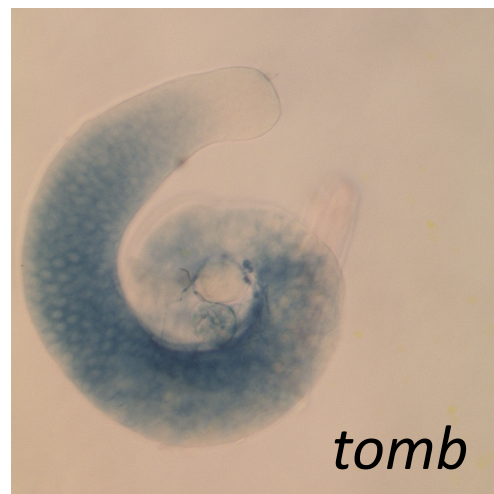

Supplement: Supplementary file 12 — Additional file 12: Fig. 9. RNA in situ hybridization localization of CG9879, Trf, and Trf2 transcripts. Upper row—in wild-type testis; lower row—in meiosis arrest mutants. CG9879 transcript is not expressed in aly mutants, while Trf and Trf2 retain their expression in mutant spermatocytes. [file 13072_2018_183_MOESM12_ESM.pdf]
